# MicroRNA-mediated bioengineering for climate-resilience in crops

**DOI:** 10.1080/21655979.2021.1997244

**Published:** 2021-12-09

**Authors:** Suraj Patil, Shrushti Joshi, Monica Jamla, Xianrong Zhou, Mohammad J Taherzadeh, Penna Suprasanna, Vinay Kumar

**Affiliations:** a Department of Biotechnology, Modern College of Arts, Science and Commerce, Savitribai Phule Pune University, Pune, India; b School of Life Science and Biotechnology, Yangtze Normal University, Ch-ongqing, China; c Swedish Centre for Resource Recovery, University of Borås, Borås, Sweden; d Bhabha Atomic Research Centre, Homi Bhabha National Institute, Mumbai, India

**Keywords:** Bioengineering, climate change, crop improvement, environmental stress, gene expression, combined stress, miRNA

## Abstract

Global projections on the climate change and the dynamic environmental perturbations indicate severe impacts on food security in general, and crop yield, vigor and the quality of produce in particular. Sessile plants respond to environmental challenges such as salt, drought, temperature, heavy metals at transcriptional and/or post-transcriptional levels through the stress-regulated network of pathways including transcription factors, proteins and the small non-coding endogenous RNAs. Amongs these, the miRNAs have gained unprecedented attention in recent years as key regulators for modulating gene expression in plants under stress. Hence, tailoring of miRNAs and their target pathways presents a promising strategy for developing multiple stress-tolerant crops. Plant stress tolerance has been successfully achieved through the over expression of microRNAs such as Os-miR408, Hv-miR82 for drought tolerance; OsmiR535A and artificial DST miRNA for salinity tolerance; and OsmiR535 and miR156 for combined drought and salt stress. Examples of miR408 overexpression also showed improved efficiency of irradiation utilization and carbon dioxide fixation in crop plants. Through this review, we present the current understanding about plant miRNAs, their roles in plant growth and stress-responses, the modern toolbox for identification, characterization and validation of miRNAs and their target genes including *in silico* tools, machine learning and artificial intelligence. Various approaches for up-regulation or knock-out of miRNAs have been discussed. The main emphasis has been given to the exploration of miRNAs for development of bioengineered climate-smart crops that can withstand changing climates and stressful environments, including combination of stresses, with very less or no yield penalties.

## 1. Introduction

Climate change is one of the greatest challenges that have emerged in this century, with impacts ranging from fluctuating weather patterns, amplifying temperature, acidifying oceans with declining corals, and imperiling ecosystems, thus impacting the vital food security. According to the Intergovernmental Panel on Climate Change (IPCC) climate change refers to ‘a change in the state of the climate that can be identified (e.g. using statistical tests) by changes in the mean and/or the variability of its properties, and that persists for an extended period, typically decades or longer’ [1]. Agricultural practices and agro-ecosystems are susceptible to climate change as variations in temperature or precipitation pattern affect the growth and maintenance of crops in a particular region thereby severely influencing crop yield and quality. Furthermore, climate change aggravates land degradation through increase in rainfall intensity, flooding, drought severity, heat stress, dry spells, wind, sea-level rise, and permafrost thaw that have been modulated by land management [2]. For instance, studies published between 1980 to 2015 have shown a decline of 21%, 40% and 10% in the yield of wheat, maize and cereal crops, respectively, at global scale due to climate change associated drought conditions. Further, with every °C rise in global temperature, yields of wheat, maize, rice and soybean decrease by 6 ± 2.9%, 7.4 ± 4.5%, 3.2 ± 3.7% and 3.1%, respectively [3–6]. In order to maintain yield throughout the course of time, farmers may be forced to change cultivation practice, the time of cultivation or even type of crops grown [7], and in this context, bioengineered crops may hold the key . The crop varieties that show resilience to challenging environments and complex climate change conditions with zero or less yield penalties are desired to sustain food security for the growing population [8].

Conventional breeding techniques have been explored to improve crop durability against specific biotic and abiotic stresses using techniques such as mutation breeding, hybridization and selection from landraces resulting in significant improvement and release of new cultivars. However, these approaches are limited by challenging environmental hurdles, long generation time making the process time consuming and inefficacious. Genetic engineering has a potential to bypass these hurdles through utilizing the modern biotechnological tools such as genomics, proteomics and transcriptomics for developing stress-tolerant crop plants with less impacts on yield. Many studies have evaluated the influence of individual or combined abiotic stress factors as reviewed by Raza et al. 2019 [8]. Though most of these studies were conducted under either single stress or under controlled laboratory environment, these cannot narrate and represent true adaptations for potential impacts of climate change under field ecosystems [9]. Furthermore, the single-gene approach is largely becoming outdated as pleiotropic effect makes the agronomic traits genetically complex, in addition to this many agronomic traits are controlled by multi-gene products or multi-gene pathways making selection of single gene difficult [10]. Therefore, keeping an eye on the projected futuristic climate changes/fluctuations and their impacts on crops, there is an urgent need to identify and explore new and effective biotechnological tools for developing climate-resilient or climate-smart crops [11].

With the discovery of microRNAs (miRNAs) and their roles in almost all facets of plant life, increasing attention has been laid on elucidating complex mechanisms of miRNA mediated transcriptional or post-transcriptional regulation of genes involved in plant responses and adaptations to stress conditions [12]. MiRNAs are a class of small, conserved, endogenous non-coding RNA (ncRNAs) species that regulate many biological processes by down and up-regulation of gene expression [13]. In plants, miRNAs pair with their respective targets imperfectly in their coding site of mRNAs/transcripts to destabilize/degrade them or inhibit protein translation [14–16]. Furthermore, miRNAs mediated gene regulation via miRNA-directed mRNA cleavage, translational repression, chromatin remodeling and epigenetic modification has also been recently explored for potential use in crop improvement [17].

MiRNAs have gained considerable attention in recent years with an increasing number of reports confirming their key role in various plant molecular processes. To appraise the international scenario on filed inventions related to the miRNA, we have mined the Google Patent search tool, with a set of queries using the keywords (‘miRNA’ ‘abiotic stress’ ‘plants’ ‘transgenics’) on 4^th^ Aug’2021. The retrieved data showed that, a total 13,651 patent applications and grants were filed so far, with China accounting for the highest numbers followed by USA, WO, EP and other countries (https://patents.google.com/). The data includes patents filed for diverse areas like miRNA and its role in drought tolerance/salt resistance/heat tolerance (CN-106434654-A, CN-103114091-A, WO-2013048255-A2), biotic stress (US-2012278929-A1, WO-2013048255-A2), plant growth and development (WO-2006034368-A2), artificial miRNAs (US-2013111634-A1), genome editing (WO-2020178099-A1), methods of developing transgenic plants for enhancing agronomic traits (US-2011197316-A1, US-2015135372-A1), heavy metal stress (CN-112662701-A, CN-106754913-A) and others. It is noteworthy here that a substantial gap was observed between the patented applications and available literature data. This survey demands integrating the miRNA biotechnological tools and scientific knowledge to develop strategies for improving plant growth and productivity under stressed environment. The increasing application of high throughput sequencing and techniques such as degradome sequencing, bisulfite sequencing, massive parallel signature sequencing (MPSS) in addition to computational tools have further assisted in identification of novel miRNAs involved in defining agronomic traits. Along with this, emerging tools such as artificial miRNAs (amiRNAs) and clustered regularly interspaced short palindromic repeats (CRISPR) based genome editing for improvement of traits and better resilience to climate change have been made possible to avoid non-targeted effects [18–22]. While surveying for the global agenda 2030 ‘Sustainable Development’, authors have also considered the key aspects of an important mission; The UN 17 Sustainability development goals (SDGs), established in the year 2016 stating ‘A blueprint to achieve a better and more sustainable future for all people and the world by 2030’ [23]. This mission targets 17 SDGs involving policy makers, academicians and agro-industries people. This review article is also an attempt to integrate multiple research views allied to the five SDGs namely; Goal 2 (zero hunger), Goal 3 (good health and well-being), Goal 9 (industry, innovation and infrastructure), Goal 13 (climate action) and ultimately the cumulative Goal 17 (partnerships for the goals).

This review aims to present the current trends in crop engineering via the miRNAs and their target genes, biogenesis and regulatory mechanisms in adaptations and response under climate conditions. Modern tools and techniques such as amiRNAs, target mimicry and emerging machine learning techniques in identifying and characterizing miRNAs in plants have been described. Strategies for up-regulation or knock-out of miRNAs of interest, successful events, challenges and future prospects are also discussed.

## 2. Brief overview of plant miRNAs

**Figure 1.**
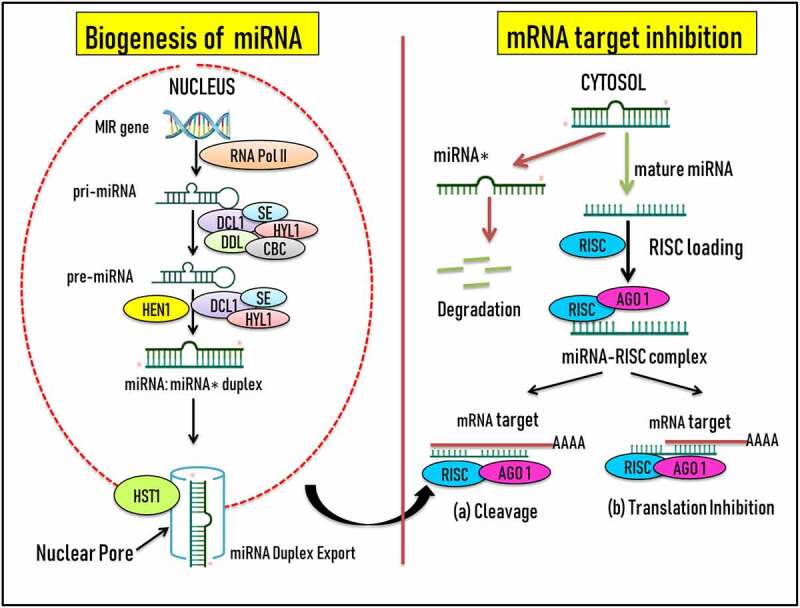
Schematic representation of the biogenesis of miRNA and modulation of target mRNAs via cleavage/translation inhibition. **Abbreviations-** DCL-1: Dicer-like RNase III endonucleases, DDL: RNA-binding protein DAWDLE, HYL1: ds-RNA binding protein HYPONASTIC LEAVES1, SE: Zinc Finger protein SERRATE, CBC: Nuclear cap binding complex, HST: Exportin protein HASTY, HEN1: HUA Enhancer 1, AGO1: ARGONAUTE, RISC: RNA-induced silencing complex

MicroRNAs (miRNAs) belonging to the class of single-stranded non-coding RNA molecules are considered to be effectual regulators of gene expression of the plant kingdom [12]. In the past several years, there has been significant research outcome on the role of small ncRNAs in the life cycle of plants growing under stressed environment [13]. Amongs these small RNA family members, panorama of miRNAs has been identified as a tiny promising pervasive class of key post-transcriptional/post-translational growth regulators often responsive to single/multiple/combinatorial stress stimuli [24,25]. First reported in *Caenorhabditis elegans*, a transparent nematode [26,27], miRNAs are single stranded 19–25nt highly conserved ncRNA molecules [12,28]. Plant-specific miRNAs were first reported by Reinhart et al. [29], adding further to the presence of evolutionary conservation across eukaryotes and prokaryotes [29]. During stressful conditions, miRNAs trigger up- or down-regulation of their target genes via four different pathways namely; mRNA cleavage, chromatin remodeling, translational repression and/or methylation of DNA [17,30]. They are synthesized by the plants during physiological, molecular, and developmental and plant growth hormone regulation processes [28]. Looking at the origin, biosynthesis and mode of action, both plant and animal miRNAs have been reported for sharing in part the similarity and differences across them. This can be further supported by the fact that several plants have more than 100 types of nuclear encoded miRNA (MIR) genes [31]. Growing evidence shows that the role of miRNAs in regulating the expression of their target genes under the given set of environmental signaling components is a tightly modulated process in both plants and animals. The regulation is intricate in the sense that single or multiple external signals can activate individual or multiple miRNAs causing differential expression of single or multiple target genes. Henceforth, miRNAs have been characterized as master regulators in response to multitude signals. Biogenesis and miRNA mode of action is a fine-tuned mechanism involving atlases of cellular and molecular switches in order to affect the dual regulation of target genes. On the basis of their genomic location, miRNAs are classified into two main types namely ‘intronic’ or ‘intergenic’. The transcription of both types of miRNAs varies; intronic types are transcribed from the introns region present in their respective host transcript whereas intergenic types are transcribed by an independent RNA Polymerase II enzyme. MIR genes are transcribed by either RNA Pol II or Pol III into primary miRNA transcripts, pri-miRNAs (Figure 1). Thereafter, pri-miRNA undergoes an imperfectly folding mechanism resulting in a double-stranded stem loop hairpin precursor RNAs, pre-miRNA. Numerous cellular proteins are known to support this folding mechanism of pri-miRNA namely, Dicer-like RNase III endonucleases (DCL-1), RNA-binding protein DAWDLE (DDL), Zinc Finger protein SERRATE (SE), dsRNA binding protein HYPONASTIC LEAVES1 (HYL1), and nuclear cap binding complex (CBC) [32]. The plant stem loop structure varies in size from 60–500 nt whilst in the animal system it is ∼70 nucleotides [33]. Also, in case of plants , the processing of pri miRNAs can happen from either the loop-proximal to loop-distant site or contrariwise [34]. Moving ahead, DCL1, HYL1, and SE assisted processing results in the formation of miRNA: miRNA*; duplex (19–25 nt) having 3ʹ overhangs at their ends [35,36]. Further, methylation occurs at only 2ʹ – OH position supported by another class of protein, small RNA methyltransferase HUA Enhancer 1 (HEN1). From here the duplex is exported from the nucleus into the cytosol with the help of an exportin protein HASTY (HST). Once into the cytoplasm, the duplex gets separated wherein, the next protein, ARGONAUTE (AGO), is ready to be assembled with the guide strand, miRNA. The other strand of the duplex, the passenger strand, miRNA*, gets degraded. RNA-induced silencing complex (RISC) mediated negative and/or positive regulation of the expression of target mRNA/mRNAs is highly relied on the complementary base pairing between the sequences present on the mRNA and miRNA respectively [12,24,37,38]. Presuming the given set of conditions, degree of base pairing is one of the pivotal factors responsible for either expression or repression of the target mRNA through four different pathways. Of note is the base pairing, which is sterner in plant systems then their animal counterparts.

Till date, there are several reports of specific plant miRNAs in response to different types of environmental stresses and changing climate conditions across diverse plant species [38–40]. Plants under stress respond concomitantly with the complex yet controlled modulation of MIR genes and it is one of the fundamental steps in miRNA biogenesis that needs to be explored at a larger perspective for better understanding of the complex stress regulatory pathways. Eventually several reports have been published showing utilization of miRNA in various crop improvement programs without impacting agronomic traits by using an array of biotechnology and bioinformatics techniques. Owing to their ubiquity and complexity, miRNAs-based climate smart crops will be the possible strategy to balance the food and nutritional health of the rising global population rate [13,41].

## 3. Identification and characterization of plant miRNAs and their targets

The hereditary information which determines the function, structure, organization and cellular responses of an organism are stored in the genome. Hence understanding the mechanistic insights of plant responses and adaptation to challenging environmental conditions via decoding the genetic sequences and their transcripts has been in the focus historically. The genome, transcriptome, proteome and metabolomics play a vital role in understanding the hidden codes [42]. The miRNAs present in an organism play regulatory roles as discussed earlier and hence their sequencing, identification and characterization are of utmost importance. The derived information can be used for imparting the necessary tolerance to plants and crops based on futuristic climatic conditions [13]. The miRNA modification would keep the base genome untouched and would rather focus on enhancing or retarding the regulatory mRNAs . A brief description of modern tools and techniques currently in use for miRNA related work is given below.

### 3.1 Deep sequencing

Next generation sequencing (NGS) or deep sequencing technologies have revolutionized the discovery [43] and identification of various novel and conserved miRNAs helping to fill the missing gaps in our knowledge. The small size of miRNAs along with their repetitive nature in the genome conferred significant challenges for sequencing. Sanger’s dideoxy synthesis method [44,45] and Maxam–Gilbert’s chemical cleavage method [46] are the founding techniques based on which the NGS came into existence. Illumina (Solexa) sequencing is one of popular sequencing platforms with its removable fluorescent labels. It is sub-classified into platforms like HiSeq, MiSeq, MiniSeq, NextSeq and NovaSeq. At 600 Gb of sequence yield per run and much greater resolution, it is significantly better in comparison with other techniques like Roche 454 pyrosequencing, Sequencing by Oligonucleotide Ligation and Detection (SOLiD) and Ion Torrent. When used for transcriptome sequencing specializing on small RNA, the Illumina NGS is a preferred choice for identifying novel miRNAs under varying environmental conditions. Third generation, an advanced version of NGS, is cheaper, faster, accurate and reliable [47]. Single-molecule real-time (SMRT) sequencing (Pacific biosciences), Helicos sequencing (genetic analysis system) and Nanopore are some examples of Third generation sequencing.

The degradome sequencing technique provides a plant profile containing miRNA along with their target mRNAs, allowing one to identify novel miRNAs and target mRNAs. It is most commonly utilized technique for degraded RNA under which sequencing and identification of ncRNAs, microRNA (miRNA), short-interfering RNA (siRNA), turnover RNA fragments, and miRNA-/siRNA-cleaved target RNA fragments can be performed. It is a result of combining modified 5ʹ rapid amplification of cDNA ends (5ʹ-RACE) and ‘NGS’ [48]. The 3ʹ RNA fragment is usually cleaved under miRNA action, and degraded by XRN4. When compared to 5ʹ-cleaved RNA fragment XRN4 exhibits a lower degradation rate. Hence quantification of 3ʹ-cleaved RNA fragments provides reliable data to demonstrate the miRNA-target relationship. Using NGS and various computational approaches the 3ʹ-cleaved RNA fragments in the total RNAs can be converted to cDNAs by 5ʹ-RACE [48].

Recently Selvi et al identified 1224 conserved miRNAs and 435 novel miRNAs from *Saccharum* under drought stress using high-throughput miRNA deep sequencing method [49]. Xu et al [50]used PacBio RSII and the Illumina sequencing platform to identify heat responsive LncRNA associated with heat shock protein in *Populus qiongdaoensis* seedlings. Pacbio sequencing technology was used to discover full length transcripts of *Pinus elliottii* for a phylogeny study [51]. 24 putative novel miRNAs were identified from a leaf of *Persicaria minor*, a non-model plant, transcriptome using 2500 HiSeq Illumina [52]. Similarly, transcriptome sequencing was done in *Jatropha curcas* by high-throughput sequencer called Illumina NextSeq500 resulting in identification of 266 conserved miRNA belonging to 78 miRNA families [53]. In *Populus*, degradome sequencing has been applied to identify cold responsive miRNA and their targets using Illumina HiSeq 2000 [54]. Identification of drought stress responsive miRNA present in *Macleaya cordata* was done by Illumina Hiseq xten Sequencer which showed presence of 355 miRNAs belonging to 68 families, miR166 being the most prominent [55]. Deep sequencing using Illumina Hiseq 2500 platform by Novogene (Beijing, China) revealed 88 conserved and 13 novel miRNAs in *Reaumuria songorica* under salt stress during seed germination [56]. With the use of high throughput deep sequencing technology, 67 conserved and 66 novel miRNAs were identified in *Gossypium hirsutum* L. in response to root-knot nematode *Meloidogyne incognita* infection [57]. In another study, the high-throughput sequencing platform was utilized to perform a transcriptome-wide analysis for the identification of miRNAs produced in response to DNA damage in rice. The study identified 513 known and 72 novel miRNAs in the roots of bleomycin-treated and control rice specimens [58]. Wu et al utilized the Illumina NovaSeq6000 by Gene Denovo Biotechnology Co. (Guangzhou, China) to identify miR9736, miR5059, miR167, miR5665, miR866, miR10186, miR8165, miR857, miR399, miR399, miR163,and miR393 that plays a key role in regulating seed germination-genes [59].

### 3.2 In silico tools and databases

Recent advancement in improving crops that can withstand changing climatic conditions is mostly based on understanding the multidimensional regulatory network that occurs in plant cells at a molecular level. The candidate genes along with their targets are often identified from a wide pool of datasets. These are then characterized structurally, functionally and physiologically by comparing them with a homologous base. High throughput techniques have been established that are faster, more reliable and efficient resulting in the need for an *omics* approach in plant stress biology [60]. The high usage of miRNAs for conferring tolerance to plants has flooded the databases with a big set of information on this subject. Hence miRNAomics have been developing at a faster rate.

Plant miRNA database (PMRD) (http://bioinformatics.cau.edu.cn/PMRD/) [61], Tarbase (http://carolina.imis.athenainnovation.gr/diana_tools/web/index.php) [62], MiRDB (http://www.mirdb.org/) [63] and miRbase (https://www.mirbase.org/) [64] are most commonly used databases that contain all published miRNAs based on which one can compare and identify novel miRNAs in plant species. miRNEST 2.0 represents a wide-ranging database of animal, plant, and virus miRNAs (http://mirnest.amu.edu.pl) [65]. Plant miRNA Target Expression Database (PMTED) (http://pmted.agrinome.org/) [66], Target prediction for plant miRNAs (TAPIR) (http://bioinformatics.psb.ugent.be/webtools/tapir/) [67] are some other databases that provide expressional details along with target prediction. Other than these databases, PlantCircNet: a database for plant circRNA–miRNA–mRNA regulatory networks [68] (http://bis.zju.edu.cn/plantcircnet/index.php) and GreenCircRNA: a database for plant circRNAs that act as miRNA decoys (http://greencirc.cn) [69] are currently used for identification of miRNAs that are interactive with cirRNA. PlanTE-MIR DB [70](http://bioinfo-tool.cp.utfpr.edu.br/plantemirdb/), is a database that harbors transposable element-related microRNAs in plant genomes that are publicly available.

The miRBase (http://www.mirbase.org/) is a most common searchable online repository that compiles all published miRNA sequences and annotations [64]. A predicted portion of hairpin structure of putative miRNA transcript is made available along with precise location and sequence information of mature miRNA. The data available on miRBase is easily downloadable and accepted for publication. As of October 2018, under release 22.1, a total of 38,589 miRNA entries have been deposited in this database. The miRNA data from 271 organisms belonging to 6 major clades (Alveolata, Chromalveolata, Metazoa, Mycetozoa, Viridiplantae and Viruses) have been made available.

Plant miRNA Encyclopedia (PmiREN, http://www.pmiren.com/) [71], is a knowledge-based database which includes processed small RNA libraries using miRDeep-P2 and a manual curation. As of now PmiREN harbors 16,422 high confidence novel miRNA loci in 88 plant species and 3,966 retrieved from miRBase. Information on precursor sequence, precursor secondary structure, expression pattern, clusters and synteny in the genome, potential targets supported by Parallel Analysis of RNA Ends (PARE) sequencing, and references is attached.

MepmiRDB (http://mepmirdb.cn/mepmirdb/index.html) [72] is a freely available medicinal plant microRNA database. The miRNA information on sequences, expression patterns and regulatory networks has been included along with organ/growth condition-specific expression information of the mature miRNAs. The ‘Interaction’ module offers the information of the degradome-validated miRNA – target pairs of eight plant species.

In recent times, due to an increase in the Small RNA sequencing procedures, a huge amount of data has been created. Hence various databases are utilized to store, characterize and keep record of all published, annotated miRNAs. A summarized list of such databases has been given in Table 1.

**Table 1.** Computational tools and databases useful for plant miRNAs and their characterization

Tools/ Database	Description	Url	Reference
**Databases specific for miRNAs**			
TarDB	Data available on plant miRNA targets and miRNA-triggered phased siRNA	http://www.biosequencing.cn/TarDB	[73]
sRNAanno	Large collection of miRNA, phasiRNA- and hc-siRNA-generating loci from approx. 140 plant species	http://www.plantsrnas.org/	[74]
IsomiR_Window	contains isomiRs and their annotations acquired from RNA-seq datasets from animals and plants	https://isomir.fc.ul.pt/	[75]
PlantCircNet	Repository of plant circRNAs–miRNA–mRNA regulatory networks	http://bis.zju.edu.cn/plantcircnet/index.php	[68]
GreenCircRNA	A database for plant circRNAs that act as miRNA decoys	http://greencirc.cn	[69]
Plant miRNA Encyclopedia (PmiREN)	Knowledge-based database which includes processed small RNA libraries using mirdeep-P2 and a manual curation	http://www.pmiren.com/	[71]
MepmiRDB	Freely available medicinal plant miRNA database	http://mepmirdb.cn/mepmirdb/index.html	[72]
miRTarBase 2020	Experimentally validated miRNA -target interactions database.	https://mirtarbase.cuhk.edu.cn/~miRTarBase/miRTarBase_2019/php/index.php	[76]
DIANA-TarBase	Catalogs published experimentally validated miRNA:gene interactions.	http://carolina.imis.athenainnovation.gr/diana_tools/web/index.php	[62]
A Rice miRNA: mRNA Interaction Resource (ARMOR)	Database of experimentally validated expression profiles of miRNAs under different developmental and abiotic stress conditions of Indian rice cultivars	http://armor.icgeb.trieste.it/login and https://www.icgeb.org/armor.html	[170]
Degradome-Based Plant MiRNA–Target Interaction And Network Database (DPMIND)	All available plant degradome data collection for retrieval and analysis of miRNA–target interactions and miRNA regulatory networks	https://cbi.njau.edu.cn/DPMIND/	[77]
Wheat MicroRNA Portal (WMP)	miRNA data compiled from published wheat microRNAs from different studies into 10 small RNA libraries under different abiotic stress.	http://wheat.bioinfo.uqam.ca/index.php	[78]
IsomiR Bank	A research resource for tracking IsomiRs	http://mcg.ustc.edu.cn/bsc/isomir/	[79]
MiRDB	Repository of miRNA target prediction and functional annotations	http://www.mirdb.org/	[63]
miRNEST 2.0	Integrative collection of animal, plant and virus microRNA data	http://mirnest.amu.edu.pl	[65]
starBase v2.0	Collection of CLIP-Seq experimentally supported miRNA-mRNA and miRNA-lncRNAinteraction networks	http://starbase.sysu.edu.cn/	[80]
Plant Non-Coding RNA Database (PNRD)	Collection of plant ncRNAs and resources, we designed an updated platform called plant ncRNA database (PNRD) based on its predecessor PMRD	http://structuralbiology.cau.edu.cn/PNRD	[81]
Plant miRNA Target Expression Database (PMTED)	Plant specific database, to study miRNA functions by inferring their target gene expression profiles from existing Microarray data	http://pmted.agrinome.org/	[66]
PASmiR	Database for miRNA molecular regulation in plant abiotic stress	http://pcsb.ahau.edu.cn:8080/PASmiR/f	[82]
Plant miRNA database (PMRD)	Integrates available plant miRNA data deposited in public databases, gleaned from the recent literature, and data generated in-house	http://bioinformatics.cau.edu.cn/PMRD	[61]
Target prediction for plant miRNAs (TAPIR)	Collection of plant miRNA targets to help in prediction.	http://bioinformatics.psb.ugent.be/webtools/tapir/	[67]
Plant microRNA Knowledge Base (PmiRKB)	Collection of miRNAs of two model plants, *Arabidopsis thaliana* and *Oryza sativa* subdivided into four major functional modules, ‘snps’, ‘Pri-miRNAs’, ‘mir – Tar’, and ‘Self-reg’.	http://bis.zju.edu.cn/pmirkb/	[83]
PlanTE-MIR DB	database for transposable element-related microRNAs in plant genomes	http://bioinfo-tool.cp.utfpr.edu.br/plantemirdb/.	[70]
miRFANs	A database for *Arabidopsis thaliana* miRNA-functional annotation	http://www.cassavagenome.cn/mirfans/	[84]
miRbase	Compiles all published miRNA sequences and annotations	http://www.mirbase.org/	[64]
**Tools specific for miRNAs and their targets**			
PAREameters	Downloadable tool for identifying computational inference of plant miRNA–mRNA targets	http://srna-workbench.cmp.uea.ac.uk/pareameters/	[106]
psRNATarget	Identifies plant sRNA targets by sequence homology and target site accessibility	http://plantgrn.noble.org/psRNATarget/home	[103]
iwa-miRNA	miRNA annotation in plant species by combining computational analysis and manual curation	http://iwa-miRNA.omicstudio.cloud/	[107]
mirMachine	Plant miRNA annotation using structured pipeline	https://anaconda.org/bioconda/mirmachine	[85]
miR-MaGiC	Performs stringent mapping to a core region of each miRNA and defines a meaningful set of target miRNA sequences taking reference from miRBase V 22.1	https://github.com/KechrisLab/miR-MaGiC	[86]
miRDeep-P2	Analysis of the microRNA transcriptome in plants. Updated version of miRDeep-P	https://sourceforge.net/projects/mirdp2/.	[87]
TarHunter	Predicts conserved microRNA targets and target mimics in plants	http://tarhunter.genetics.ac.cn/	[88]
miRnovo	Prediction of microRNAs from small RNA sequencing data, with or without a reference genome	http://wwwdev.ebi.ac.uk/enright-dev/mirnovo/	[89]
isomiR2Function	Identifies MicroRNA Variants (isomiRNAs) in Plants from any miRNA-seq profiling study along with identification of the templated and non-templated 5′- isomiRs and 3′- isomiRs.	https://github.com/347033139/isomiR2Function.	[90]
miRNA-Truncation and Tailing Analysis (miTRATA v1.3)	Truncation and tailing analysis of miRNA that is used to analyze 3 modifications	https://wasabi.ddpsc.org/~apps/ta/	[105]
miRNAFold	*Ab initio* miRNA prediction in genomes by identifying microRNA precursors and predicting microRNA hairpin structures.	https://evryrna.ibisc.univ-evry.fr/miRNAFold	[91]
isomiR-SEA	RNA-Seq analysis tool for miRNAs/isomiRs expression level profiling and miRNA-mRNA interaction sites evaluation	https://eda.polito.it/isomir-sea/	[92]
Mirpath	Identifies targets based on predicted miRNA targets (in CDS or 3ʹ-UTR regions) provided by the DIANA-microT-CDS algorithm or experimentally validated miRNA interactions derived from DIANA-TarBase	http://snf-515788.vm.okeanos.grnet.gr/	[93]
Multiple instance learning of Binding Sites of miRNA TARgets (MBSTar)	Tool used for prediction of true or functional miRNA binding sites	http://www.isical.ac.in/~bioinfo_miu/MBStar30.htm.	[94]
mTide	An integrated tool for the identification of miRNA-target interaction in plants	http://bis.zju.edu.cn/MTide/	[95]
miRPlant	Predicts novel plant miRNA from 16 plant miRNA datasets from four different plant species	https://sourceforge.net/projects/mirplant/	[96]
miRanalyzer	Detection of known and prediction of new microRNAs in high-throughput sequencing experiments of 6 plant species	http://bioinfo2.ugr.es/miRanalyzer/miRanalyzer.php.	[97]
miRExpress	*In silico* generation of miRNA expression profiles from high-throughput sequencing of RNA data	http://miRExpress.mbc.nctu.edu.tw.	[98]
PITA	Structural identification of miRNA targets thermodynamic promotion or disfavoring the interaction.	https://www.mybiosoftware.com/pita-6-microrna-prediction-tool.html	[99]
RNA22 version 2.0	Tool used to identify MicroRNA binding sites and their corresponding heteroduplexes via Interactive, Pre-computed and Full sets prediction	https://cm.jefferson.edu/rna22/	[102]
microInspector	Detection of miRNA binding site by cross referencing against known datasets	http://bioinfo.uni-plovdiv.bg/microinspector/	[104]
MiRAlign	Genome-wide computational approach to detect miRNAs in animals and plants (*Arabidopsis thaliana* and *Oryza sativa*) based on both sequence and structure alignment	http://bioinfo.au.tsinghua.edu.cn/miralign	[100]

### 3.3 Identification of miRNAs and their targets

Identification of miRNAs can be done via experimental and computational approaches. The initial attempts started in the early 2000s when Llave et al. [101] and Reinhart et al.[29] identified miRNA using direct-cloning approaches. The advancement in computational approaches jumpstarted their identification along with the target genes these miRNAs degrade. Several computational tools and software have been made available in recent times that predict the targets of miRNAs using well established databases as mentioned earlier [38].Most of the programs use different steps including to acquire data and predict/finding the targets, the base-pairing pattern of miRNA and 3ʹUTR of target mRNA sequence, the miRNA-mRNA duplex thermodynamic stability and third, the evolutionary conservation of miRNA-target sites in different species. RNA22 [102], PITA (https://www.mybiosoftware.com/pita-6-microrna-prediction-tool.html), psRNATarget (http://plantgrn.noble.org/psRNATarget/home) [103], and microInspector (http://mirna.imbb.forth.gr/microinspector/) [104] are some of the commonly used tools for finding targets of identified miRNAs.

The miRNA-truncation and tailing analysis (miTRATA) uses truncation and tailing analysis of miRNA that is used to analyze 3 modifications [105]. PAREameters (http://srna-workbench.cmp.uea.ac.uk/pareameters/) is an offline downloadable tool for identifying computational inference of plant miRNA–mRNA targeting rules using small RNA and degradome sequencing data. It is used to identify and quantify the differences between subsets of miRNA–mRNA interactions in model and non-model organisms [106]. Iwa-miRNA is a Galaxy-based framework that can be utilized to annotate miRNAs by computational analysis and manual curation. It generates a comprehensive list of candidate miRNAs from known databases and predicts novels sets from sRNA-Seq datasets [107].A summarized list of tools along with their descriptions has been given in Table 1.

## 4. Approaches for developing miRNA-mediated bioengineered crops

### 4.1 Transgenesis, cisgenesis and intragenesis

With the advancement of transformation techniques, a new arena has opened up where the MIR genes could be transferred within and between the various crossable and non-crossable plant species. Transgenesis (transfer of one or more MIR gene between non-crossable plant species), cisgenesis (transfer of MIR gene between crossable species) and intragenesis (transfer of MIR gene between same species) utilize the T-DNA of *Agrobacterium* and engineer it to carry the gene of interest for further (and desired) expression [108,109]. Cisgenesis, as described by Schouten et alis the modification done in the genetic background of a recipient plant that is cross compatible with the donor plant. It is a faster method when compared with traditional breeding wherein desired genes are introduced rapidly without any changes in other characteristics of the plant [110]. Rommens et al. [111] introduced intragenesis transformation allowing customized cassettes designing to transform plants that are cross compatible. Intragenesis transformation utilizes additional promoter and termination sequences that overcome the challenges faced under overexpression of MIR genes [109].

### 4.2 Topical delivery of miRNA (pre- and mature-miRNA)

Topical delivery can also be called a transgene-free approach which was recently optimized from the topical delivery of nanostructured dsRNA molecules in model and/or crop plants. Although the technology is still in its developing phase for transfer of pre-miRNA and miRNA, the higher stability and internalization capacity of pre-miRNA creates possibilities of using this technology for transgenic free manipulation of transcriptional profile of plant. This system uses an organic (ribonucleoprotein, cross linkers) or nanoparticle (biopolymers of chitosan, silicon, carbon and clay nanosheets) carrier to deliver the internalized RNA in plant cell [112–114].These pre-miRNAs are structured like viroids flanked by pH-dependent ribozymes, engineered such that they are not processed by the plant RNAi machinery but interact with insect digestive tract cells [109]. A topical delivery of miRNA of 22 nt was done in two species namely *Nicotiana benthamiana* and *Amaranthus cruentus* to induce biogenesis of secondary transitive miRNA that cleaved the targeted mRNAs. The delivered miRNA was able to silence green fluorescent protein (GFP) transgene and 3 endogenous genes: magnesium chelatase subunit I (CHL-I), magnesium chelatase subunit H (CHL-H), and GUN4 in treated leaves of *Nicotiana benthamiana* [115].

### 4.3 Artificial miRNA and target mimicry

To overcome the problem of nonspecific gene silencing, a side effect of siRNA-based gene silencing, artificial miRNAs (amiRNAs) were developed [116]. These amiRNAs are highly efficient, specific and are proven to have more potential to silence undesirable gene expression. The desired gene is designed and integrated into a stem-loop backbone forming a precursor (which helps in *in vivo* processing of amiRNA) resulting in decreased accumulation of mature amiRNAs in plant cells [117]. Thus the original sequence structured as miRNA-5p:miRNA-3p is replaced with a bioengineered sequence that is specific for inhibiting target mRNA. A significant feature, processing of pre-amiRNAs to form mature amiRNAs, makes them very specific in nature and avoids off-target interactions [118]. These mature amiRNAs are created for known targets, have restricted systemic movement, stable in nature and limited production of secondary siRNAs from pre-amiRNA strands [109].

Target mimicry is often utilized to regulate the biological processes in plants that are induced due to different plant behaviors. This process can be divided into two types based on their origin, endogenous target mimicry (eTMs) and artificial. The eTMS include actions of long noncoding RNA (lncRNA) and circular noncoding RNA (cirRNA) that’s been transcribed from the plant genome under varying stress conditions. Similar in function, artificial short tandem target mimic (STTM) was developed that modulated the accumulation of miRNAs. The STTMs are expressed transiently or constitutively based on the promoter (induced or tissue specific) they are built with. Some are engineered with high nucleotide sequences on an lncRNA basal template that identify and target mRNA [119]. Another class of artificial target mimicry includes miRNA SPONGES that are produced from transgenes containing multiple miRNA binding sites located in tandem repeats. These SPONGES are utilized to target and inhibit whole families of related miRNAs [120,121]. An artificial *Vitis vinifera* miRNA319e (amiR319e) was introduced in transgenic *Nicotiana benthamiana* using overlapping long primers and was able to silence pre-inserted green fluorescent protein (GFP) gene [122]. Von et al. [123], in their comparative study conducted on *Arabidopsis thaliana*, showed that the efficiency of amiRNA declined based on their environmental conditions and developmental stages of plant as compared to miRNAs. The research showed amiRNAs were unable to silence the targeted gene with age during its vegetative growth stage in a temperature dependent manner.

### 4.4 MIR gene editing

A sequence specific editing is required for specific and efficient modulation of a particular gene. This genome editing tool involves modulation of a gene that is inherently present inside the plant and no integration of foreign DNA into the host plant is conducted [124]. There are four types of editing tools used for MIR gene modification: (1) zinc finger nucleases (ZFN), (2) meganucleases, (3) transcription activator-like effector nucleases (TALENs), and (4) CRISPR systems [125]. By using basic cellular repair mechanism like non-homologous end-joining (NHEJ) and homology-directed recombination (HDR) pathways for repairing the double stranded breaks caused by these endonucleases, allows the insertion and deletion (INDELs) of desired gene [126].

The CRISPR-associated protein-9 nuclease (CRISPR/Cas9), is the most popular, modern, simplest Nobel Prize winning (https://www.nobelprize.org/prizes/chemistry/2020/press-release/) method of genome editing. CRISPR/Cas9 works by retarding the miRNA biogenesis through introduction of INDELs at miRNA processing sites of MIR genes [127,128]. Apart from biogenesis, the INDELs can also hamper miRNA-mRNA target pairing, full deletion and knock-in of the MIR genes and/or their promoters [20]. CRISPR-derived systems, such as dCas9 nickase79, fCas980, Cpf181, Cpf1 and Csm1 are being searched and studied for more flexible, efficient and applicable usage [129].Despite being most used, CRISPR/Cas9 faces some complexities and challenges while editing MIR genes. MIR genes have highly complex regulatory networks which makes knock-down or knock-in experiments harder to conduct. Along with this, the smaller length of MIR genes requires shorter guide RNA (gRNA) reducing the number of possible gRNA/Cas9 targets thus reducing possible hits on mature miRNA. Furthermore, CRISPR/Cas13a which uses novel nuclease type (class II type VI-A endoribonuclease) is under study but has not yet been established for pre-, pri-miRNA or mature miRNA editing in plants [109]. Recombinant codon-optimized Cas9 (rCas9) was prepared for rice with the use of single-gRNA that were specifically targeting mature miRNA sequences or sites required for the biogenesis of mature miRNA. 409 transgenic plants of rice were formed in a CRISPR/Cas9 mediated mutation on 13 independent miRNAs involved in drought tolerance responses [130].

## 5. Exploring machine learning and/or artificial intelligence for miRNA research

Machine learning (ML) or artificial intelligence (AI) is a discipline that uses computational and statistical approaches to predict from unseen data based from experiences learned from previous experiments. These ML algorithms learn from a set of known labeled data to make future predictions, thus reducing the false positives and increasing the specificity of the ML models [131]. Feature selection is one of the fundamental problems faced by ML during ranking of miRNAs. When it comes to prediction of miRNAs, ML has been used to determine stress responsive miRNAs wherein it learns the complex non-linear patterns between the miRNA expression (input) and plant stress (output). Supervised learning methods like decision tree (DT), support vector machines (SVMs), least-square support vector machines (LSSVMs), and naïve Bayes (NB) can be used to find the patterns in a database [132]. The ML prediction of novel miRNAs has a high-class imbalance because fewer well-known miRNAs are being compared with the whole genome of the candidate plant. When the known dataset is prepared in a manner where the model, experiments and performance are not properly addressed, the pre-miRNA prediction works in a biased and nonspecific manner. Hence the labeling of positive as well as negative datasets are of utmost importance [133].

Bugnon et al. [134] used ML to conduct a genome wide identification of pre-miRNAs. The research study included comparison of genome of five model organisms (*Arabidopsis thaliana, Caenorhabditis elegans, Anopheles gambiae, Drosophila melanogaster, Homo sapiens*) with known pre-miRNAs as their labeled dataset. The study concluded that for smaller genomes and imbalances, all methods perform in a similar way but for larger datasets such as the *Homo sapiens* genome, deep learning approaches using raw information from the sequences reached the best scores. PlantMiRNAPred (compares all miRNA plants in miRBase as positive and Pseudo hairpins from the protein coding sequences of *Arabidopsis thaliana* and *Glycine max* as negative) [135], miRPara (dataset made from animals, plants and virus in miRBase and sequences with pri-miRNAs as positive and negative selection respectively) [136] and SMIRP (Species-specific positive sets from miRBase and ncRNA as negative set) [137] are some of the ML approaches available for novel miRNA prediction in plants [133]. Thus, though the use of ML and/or AI for plant miRNA work is still in infancy and far from full potential exploration, these initial attempts are very encouraging and are expected to attract more studies that will help their exploration further.

## 6. MiRNA-based biotechnology for developing bioengineered climate resilient crops

**Figure 2.**
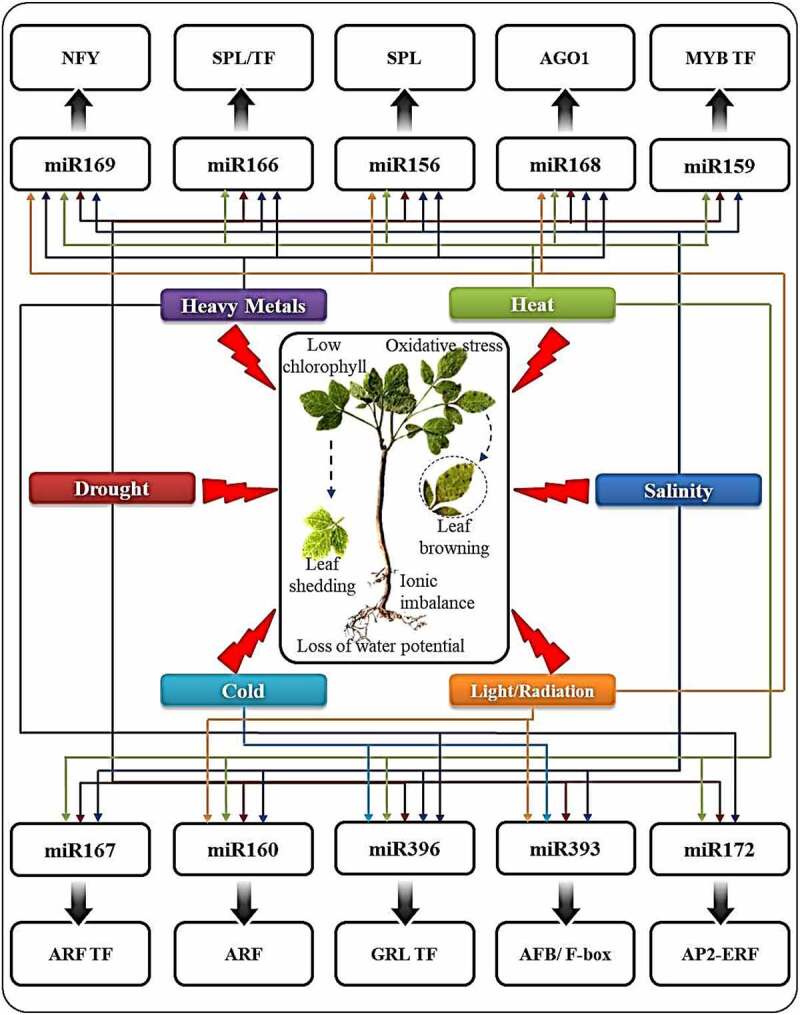
Schematic representation of the miRNAs responsive to multiple abiotic stress factors and their respective targets. **Abbreviations-** NFY: Nuclear Factor Y, SPL: SQUAMOSA Promoter-binding Protein-Like, TF: Transcription Factor, AGO1: Argonaute, MYB: Myeloblastosis, ARF: Auxin Response Transcription Factor, GRL: Growth Factor-Like, AFB: Auxin-binding F-Box AP2: APETALA2. Reproduced with permission from Xu et al. 2019, Copyright 2019, Elsevier [13]

Challenging or unfavorable environments are known to confer negative impacts on plant growth, development and yield. The expression levels of key genes are often altered in such stress conditions due to differential regulators, transcription factors and miRNAs expression as part of the plant’s responsive mechanism. Numerous miRNAs have been identified, characterized and used for transgenic plant development in response to confer tolerance to various abiotic stresses [13,28].Along with singular stresses, the plants often face multiple stress factors in nature, or more specifically in agro-ecosystems. The environment mimicry is thus attempted in contemporary times via studying the impact of combinational or multi-factorial stresses as well as sequential stresses which are seen to cause more than 50% of crop loss worldwide [138,139]. Recent reports confirm that the sound platform miRNAs can be used for engineering crops that can withstand the singular as well as multiple environmental stresses, or climate changes [13]. Figure 2 depicts recently identified miRNAs from crops involved in plant responses to multiple environmental challenges.

### 6.1 Individual stresses

#### 6.1.1 Drought stress

Water availability is an important factor in plant growth and development processes and thus drought stress in considered a major challenge for optimal crop production and yield. Changing precipitation rate due to increase in global warming has changed the seasonal pattern of rainfall. In the near future there is a possible shift in the EI Nino-Southern Oscillation (ENSO) and Indian monsoon rainfall due to global warming [140]. Similar observations were done regarding the East Asian summer monsoon (EASM) rainfall under global warming based on the historical and representative concentration pathway (RCP) [141]. Hence there is an intermittent drought, and flash floods that damper the crop growth, along with hampering the soil physiology.

Hajyzadeh et al. [142] created transgenic chickpea line overexpressing miR408 to examine its role in drought conditions. The study reported overexpression of miR408 caused reduced plastocyanin transcript expression which resulted in dehydration-responsive element-binding protein (DREB) regulation under drought conditions conferring enhanced tolerance as compared to the wild-type line. Similarly, Hang et al. [143]worked on miR408 of *Oryza sativa* (*Os-miR408*) to develop transgenic *Lolium perenne* (perennial ryegrass) line to confer tolerance against drought. The transgenic perennial ryegrass showing heterologous expression of *Os-miR408* exhibited higher leaf relative water content (RWC), lower electrolyte leakage (EL) and less lipid peroxidation resulting in improved drought tolerance as compared to wild-type plants, under drought stress. The results suggested that the improved drought tolerance in *Os-miR408* transgenic plants could be due to leaf morphological changes favoring the maintenance of water status and increased antioxidative capacity protecting against the reactive oxygen species damages that occurs under such conditions [143]. Similarly, transgenic *Hordeum vulgare* (barley) overexpressing *Hv-miR82* under drought conditions conferred drought tolerance to plant when the transgene was regulated by a drought induced promoter [144]. Visenti et al. [145] compared wild-type and transgenic tomato overexpressing miR156-oe under drought conditions with strigolactone-depleted and strigolactone-treated plants. The study showed *miR156* overexpression and strigolactone treatment led to lower stomatal conductance and higher ABA sensitivity conferring faster drought recovery. MiR156 has also been reported to play a key role in conferring drought tolerance by its interaction with SPLs (MsSPL6, MsSPL12, and MsSPL13) in *Medicago sativa* [146].

In contrast, Zhou et al. [147] reported that *gma-miR398c* negatively regulated the peroxisome-related genes (*GmCSD1a/b, GmCSD2a/b/c* and *GmCCS*) under drought conditions. When *gma-miR398c* was overexpressed in transgenic *Arabidopsis thaliana*, it resulted in reduced survivability under water deficient condition, sensitivity to drought during seed growth and germination leading to decline in percentage germination rate along with higher rate of water loss in leaf. Similarly, when *gma-miR398c* was overexpressed in transgenic *Glycine max*, the expression levels of *GmCSD1a*/b, *GmCSD2a*/b/c and *GmCCS* were decreased phenomenally as compared to the knocked out *miR398c* and wild-type soybean under drought conditions [147].

#### 6.1.2 Salinity stress

Hyper soil salinity is a secondary effect of irrational irrigation practices, improper drainage, overuse of fertilizers as well as the climatic changes. The predicted increase in the temperature and decline in precipitation leads to drying of soil, inducing an increase in the ionic concentrations [148]. Besides, the global warming is projected to rise the sea water levels causing increased infusion of salinity within the inland soil [139]. The salt stress dramatically affects the crop growth and yield resulting in low harvest, poor quality of seed and less availability of food from the agricultural sector [149].Ma et al. [150], analyzed miR156/SPL from *Malus domestica* (apple) which is seen to regulate embryogenesis, morphogenesis, life cycle stage transformation, flower formation and other processes. The overexpression of *MIR156a* induced salt sensitivity in apples, whereas,*MdSPL13*overexpression stabilized the tolerance levels.MiRNA408 is an abiotic stress responsive candidate and is reported to be involved in conferring salinity tolerance in *Nicotiana benthamiana* [151]. Overexpression of *Sm-MIR408* (the miR408 precursor sequence) and its promoter sequence in tobacco promoted seed germination, reduced the accumulation of reactive oxygen species by increasing the expression of *NbSOD, NbPOD* and *NbCAT* under salt stress [151]. A transgenic BRRIdhan 28 (BR28) (rice cultivar) was developed with DST_artificial miRNA (Drought and Saline tolerance; DST_amiRNA). Transformed plants showed vigorous growth with longer panicle length and higher primary branching resulting in higher yield under saline stress [152].

The miRNAs also known as negative regulators of stress responses including salinity stress, and their overexpression can make the plant stress sensitive. For instance, using the CRISPR/Cas9 knockout system, inhibiting *OsmiR535* in transgenic rice enhanced the tolerance to salt along with other stresses. The overexpression of *OsmiR535* reduced seedling setting under stress conditions [153]. Wang et al. [146] studied the interaction of *miR414c* with iron superoxide dismutase gene *GhFSD1* in *Gossypium* under saline stress. In cotton the *ghr-miR414c* targets the coding sequence region of GhFSD1, thus inhibiting its expression. The patterns were proven when transgenic *Arabidopsis thaliana* conferred salinity stress tolerance under ectopic expression of *GhFSD1* and salinity sensitivity when *ghr-miR414c* was constitutively expressed. The overexpression of *ghr-miR414c* in cotton decreased the expression of *GhFSD1* similar to *GhFSD1*-silenced cotton [146].

#### 6.1.3 UV light

Light is the first element sensed by a plant when it emerges from the soil. Being the inducer of various metabolic activities, its mechanism of induction is utmost necessary. That being said, its action and interaction with miRNAs is a lesser known area of research [154]. The ultraviolet (UV) radiations are usually deflected by the earth’s stratospheric ozone layer. A slight change in its thickness would create a strong biological effect due to the radiation emitted by the rays. With changing climate and increasing global warming, although unpredictable, there would be a slight change in the ozone layer thickness in the near future [155]. The role of *BrmiR828* in the light-induced synthesis of anthocyanin in *Brassica rapa*was explored by Zhou et al [156]. The study observed that light-induced down-regulation of *BrmiR828* can target BrTAS4, BrPAP1 (Bra039763), MYB82 (Bra022602) by repressing their expression levels leading to the accumulation of MYB transcription factors that confer positive regulation of anthocyanin biosynthesis in light-exposed seedlings of *Brassica rapa* [156]. Li et al. [157] showed action of LONG HYPOCOTYL 5 (HY5) on light regulated transcription of miR163 in *Arabidopsis thaliana* seedlings. Overexpression of *miR163* in transgenic *Arabidopsis thaliana* showed inhibited primary root elongation due to inhibition of its target PXMT1 under light conditions [157]. Thus, though there are only a handful of studies, the results are encouraging that miRNA-biotechnology can play a key role in developing bioengineered crops that can survive under harsh UV-radiations.

#### 6.1.4 Heavy metals

Heavy metal availability in soil has been predicted to increase with changing climatic conditions, leading to higher rates of contamination and environmental concern [158,159]. It is particularly concerning considering the high environmental health risk related with the elements, such as Lead (Pb), Arsenic (As), Mercury (Hg), Cadmium (Cd) etc. The source of contamination is not only limited to the presence of such metals in soil, but their high concentration in the air through automobile exhausts [160–162].Ding et al. [163], analyzed the role of miR166 during Cd accumulation in rice. The study observed, overexpression of *miR166* improved tolerance against Cd in transgenic rice by reducing the translocation of Cd from roots to shoots. The class-III homeodomain-Leu zipper (HD-Zip) family proteins along with HOMEODOMAIN CONTAINING PROTEIN4 (OsHB4) gene (*Os03g43930*) are usually up regulated during Cd stress, but were observed to be down regulated under overexpressing *miR166* [163]. Similarly, Shen et al. [164] worked on overexpressing *IamiR-4-3p* from *Ipomoea aquatic* in transgenic *Arabidopsis thaliana* against Cd stress. It was observed that expression level of GST3 was reduced by 20%, shoot MDA and H_2_O_2_ were higher in concentration along with shorter root and shoot length in transgenic *Arabidopsis thaliana* [164].Another study showed grafting of *Solanum lycopersicum* onto *Solanum melongena* reduces Cd accumulation in tomato by increasing the miR395b expression levels that target Phavoluta (PHV), Revolute (REV), Class III (HD-Zip), Sulfate transporter 2;1 (SULTR2;1), ATP sulfurylase (APS) (APS1/3), and High-affinity sulfate transporter (HAST) genes, which are directly or indirectly responsible for sulfate transport [165]. The study reaffirms the correlation between Cd and sulfur accumulation in tomato scions. Two Cd responsive miRNAs, miR172b-3p and miR398-3p targeting copper chaperone for superoxide dismutase (ATCCS) and Fasciclin-Like Arabinogalactan-protein 9 (FLA9) respectively, were down-regulated in leaves of *Brassica juncea* under Cd stress [166]. The study observed bra-miR172b-3p as a negative regulator that enhances inhibition of resistant responses against cd stress.

### 6.2 Combined stresses

Plants repetitively face environmental stresses whether in individual/multiple/combined form which is drastically reducing the fertile land and thus heightening the issues of loss in agricultural productivity . Looking at the preponderance of the evidence from the recent scientific literature, the niche of miRNAs is widespread in modulating the differential expression of stress responsive target genes, in particular, to the individual or multiple stresses across biota [167–169]. However, till date the role of miRNA in plant growth and development during the combined stresses are not well understood and requires more studies. Identification and understanding the up/down-regulation of miRNAs to the combination of different stresses can be a challenging task but it is a new window for proper utilization of the biotechnological tools [38,109,170,171] in developing miRNA-mediated climate-resilient futuristic crops [172]. In view of the above scenario, there are few reports utilizing various advent transgenic approaches to untangle the complex and multitude expression patterns of miRNAs and mRNAs in response to combined abiotic stresses in plants. Transgenic tobacco plant was generated expressing *Zea mayszm-miR156*for enhanced drought and salt stress tolerance using either cauliflower mosaic virus or stress inducible promoter [173]. In another study, overexpression of *Glycine maxmiR172c* was attempted by engineering *Arabidopsis thaliana* plants in response to water deficit and salt tolerance. The resulting data identified *gma-miR172c* as a positive regulator in response to the combined stresses [174]. In another genetic engineering approach using CRISPR/Cas9 knockout system technique, expression of *Oryza sativa* miR535 was studied in response to salt, drought, polyethylene glycol (PEG) and abscisic acid (ABA) stresses. It was shown that either knockout/inhibition results in tolerance of rice plants to the combined stresses and thus making it as a negative regulator of target gene expression in stressed rice plants [153].

### 6.3 Climate change

Changing climate has emerged by inflating CO_2_ concentration causing greenhouse effect and subsequently increasing global ambient temperature. Atmospheric CO_2_ concentration is predicted to rise 550–700 ppm by 2050 and to 650–1,200 ppm by 2100, which will subsequently result in a global climatic warming of 1.1 to 6.4°C by the end of this century [175]. Based on predicted climate change there will be a shift in the temperature and precipitation zones from the tropical area toward the polar caps, which will lead to an increase in crop yield in North-Western Europe and decrease the crop yield in the Mediterranean area [8]. Therefore, for creating future climate proof crops it is essential to develop crops that sustain at elevated CO_2_ and variable temperature. Chilling and heat stress have generally negative impacts on yield and growth on crops. Heat stress causes an excessive increase in membrane fluidity, a disruption of protein function and turnover, and metabolic imbalances. Plant cells sense and respond to this change in ambient temperature using various molecular mechanisms and signaling pathways [176]. miRNA induced cellular regulation is one such prominent mechanism currently widely manipulated for developing temperature resilient crops [13]. In one such study, comparing *miR156* overexpressing alfalfa plants with empty vector control under heat stress of 40°C revealed altered protein expression patterns under heat stress. Furthermore,*miR156OE* plants showed stress tolerance characteristics with unique protein expression patterns related to metabolism, photosynthesis, stress-response and plant defenses [177]. Chilling and heating stress studies demonstrated the role of miR319 and its target *GAMYB-like1* in tomato, potato and *Solanum habrochaites* [178–180]. Overexpression of *sha-miR319d* by Shi et al [181]conferred chilling and heat stress tolerance in tomato. Under stress conditions, *sha-miR319dOE* lines showed enhanced stress tolerance, including lower relative electrolyte leakage, malondialdehyde concentration, O_2_^−^ generation as well as H_2_O_2_ concentration while higher chlorophyll contents and Fv/Fm values than wild-type plants. Furthermore, *sha-miR319dOE* lines showed curly leaf phenotypes and suppressed plant height suggesting potential role of miR319d in plant growth and development.

Along with overexpression strategies, synonymous mutation, amiRNA and knockdown strategies for miRNA mediated thermotolerance have also been used for crop improvement. For instance, Zhao et al. [182] carried out a synonymous mutation in Growth Regulating Factor (GRF) 15 at the target site of *miR396a* in poplar that showed enhanced heat tolerance as well as increased photosynthesis. Transgenic *Pag*GRF15 overexpression plants were created using primer mutation of six nucleotides at the sites targeted by miR396 in such a way that it won’t hamper the encoded amino acids sequence. Transgenic plants showed significantly higher transcript as well as protein levels of GRF15 than in the wild-type plants with significantly greater tolerance to heat stress and higher values of instantaneous WUE (photosynthesis/transpiration) throughout the course of the HS treatment. In parallel, under heat stress expression of miR160 and its precursors is also considerably increased with decrease in miR160 targets, ARF10, ARF16, and ARF17 [183]. To investigate role of miR160 and its targets in *Arabidopsis thaliana* under heat stress, Lin et al. [184] created transgenic lines of artificial miRNA (MIM160) that mimics an inhibitor of miR160 miRNA, T-DNA insertion mutants of miR160 targets and overexpressing miR160 precursor a (160OE). It was demonstrated that miR160OE lines showed improved seed germination and seedling survival under heat stress while those of MIM160 had reduced adaptation to heat.

**Figure 3.**
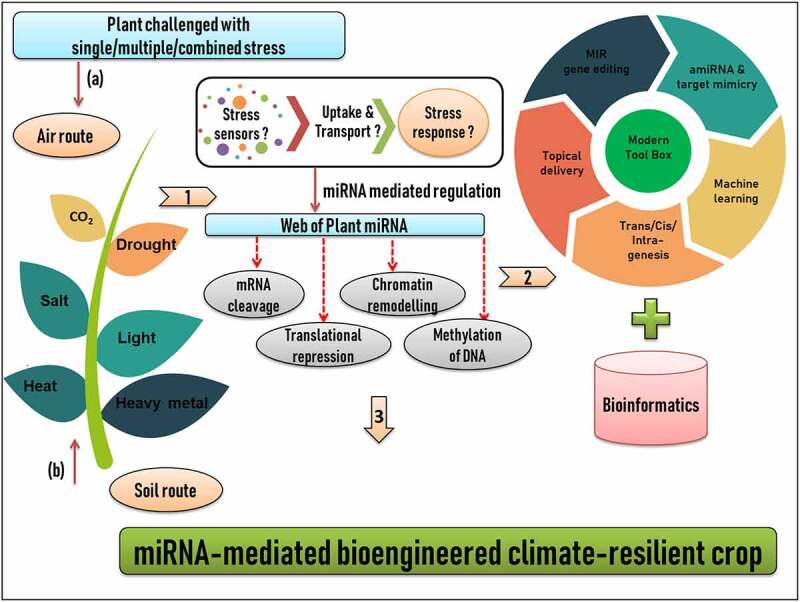
Workflow of developing miRNA-mediated bioengineered climate-resilient crops. (1) Plant growing in diverse stress environment conditions. In general, there are two common routes by which stress stimulus can reach the plant system; (a) Air route and (b) Soil route. Stress stimulus is sensed, perceived, uptake and transport by a complex set of cellular and molecular soldiers, which still demands for a critical mining (2) Identification and characterization of plant miRNAs using a technical blend of modern tool box and bioinformatics (3) Candidate miRNAs can be engineered for developing climate smart crops

In plant systems, ambient CO_2_ (Ca) uptake by stomata depends on the difference in concentration of ambient and internal CO_2_ (Ci) level and on stomatal conductance [185]. Therefore, elevated CO_2_ levels due to climate change might raise photosynthetic rate and plant primary net production or biomass. However, in open chamber experiment with elevated CO_2_ and temperature, it was seen that grain yield increased but at the cost of decrease in concentration of grain NKP and protein content, although it has also been seen that C4 plants are less responsive to elevated CO_2_ than that of C3 plants [186]. Hence it is imperative to develop crops that are better in fixing ambient CO_2_ along with increasing yield and quality to tackle yield and nutrient loss due to climate change. In this context, miRNA miR408 was overexpressed to improve photosynthesis in *Arabidopsis thaliana*, rice and tobacco [187]. Overexpression resulted in increased copper content in the chloroplast, elevated abundance of plastocyanin, and an induction of photosynthetic genes. Along with this, *miR408OE* line also showed improved efficiency of irradiation utilization and the capacity for carbon dioxide fixation. Similar studies of overexpressing and/or down regulating miRNA for improving crop tolerance to climate change are cited in Table 2, while Figure 3 gives a workflow of developing miRNA-mediated bioengineered climate-resilient crops.

**Table 2.** Plant miRNAs and their exploration for engineering crops for conferring single and combined environmental stress tolerance

miRNA	Stress	Strategy	Transgenic plant	Target genes	Responses	Reference
miR408	Drought	↑	*Cicerarietinum L*	Plantacyanin transcript	Indirect regulation of DREB and other drought responsive genes conferring tolerance against drought	[142]
	Salt	↑	*Nicotianabenthamiana*	NbSOD, NbPOD, and NbCAT	Promoted seed germination and reduced the accumulation of reactive oxygen species under salt stress.	[151]
	Drought	↑	*Loliumperenne L*	LpSOD, LpPOD, and LpCAT	Maintaining higher leaf relative water content (RWC), lower electrolyte leakage (EL) and less lipid peroxidation exhibiting drought tolerance	[143]
	CO_2_ Fixation	↑	*Oryza sativa*	OsUCL8	Cleavage of OsUCL8 by miR408 affects copper homeostasis in the plant cell, which, in turn, affects the abundance of plastocyanin proteins and photosynthesis in rice, increase in the number of panicle branches and had slightly longer grains	[188]
miRNA414c	Snalt	↑	*Arabidopsis thaliana*	Iron superoxide dismutase gene (GhFSD1)	Overexpressing gh-miR414c decreased the expression of GhFSD1 and increased sensitivity to salinity stress, yielding a phenotype similar to that of GhFSD1-silenced cotton acts as negative regulator	[189]
miR156	Drought, Salt	↑	*Nicotianatabacum cv. xanthi*	NtSPL2, NtSPL9, CP1, CP2, and SAG12	Better growth, biomass production and higher antioxidant activity	[173]
	Drought	↑	*Solanumlycopersicum*	SPL factors in tomato	The post trauma of drought which causes late reopening of stomatal opening was enhanced by miR156 in a strigolactones dependent manner. Enhances plant revival after drought stress application.	[145]
	Salt	↑&↓	*Malusdomestica*	MdSPL13	Weakened salt resistance by cleaving the target.Overexpression of MdSPL13 with knockdown of miR156 conferred tolerance in the transgenic line. Acts as a negative regulator.	[150]
	Drought, Salt	↑	*Medicago sativa* L	MsSPL6, MsSPL12, and MsSPL13	Showed decrease in height but more branches and leaves with improved salt and drought tolerance.	[146]
miR535	Drought, Salt	↓	*Oryza sativa*	OsSPL7/ 12/16	Enhance the tolerance of plants by negatively regulating the tolerance response.	[153]
miR827	Drought	↑	*Hordeumvulgare L.*	SPX, NBS-LRR domain and APT1	Enhanced leaf level stomatal conductance and photosynthetic assimilation	[144]
miR169c	Drought	↑	*Arabidopsis thaliana*	AtNFYA1, AtNFYA5, AtRD29A, AtRD22, AtGSTU25 and AtCOR15A.	more sensitive to drought stress, with reduced survival, accelerated leaf water loss, and shorter root length than the wild-type plants. Acts as negative regulator	[190]
miR1508a	Drought	↑	*Glycine max* L.	Pentatricopeptide repeat (PPR) genes and growth related genes	Dwarfing, thick cell walls, lower survival rates and greater leaf water loss was observed. Negative regulator.	[191]
	Cold				exhibited cold tolerance at the germination and young seedling stages along with higher soluble sugar content. Positive regulator.	
miR1916	Drought	↑	*Solanumlycopersicum*	histone deacetylases (HDAC) and	Negatively affected the osmoregulation and increased ROS accumulation.Negative regulator	[192]
			and *Nicotianatabacum*	strictosidine synthase (STR)		
miR393	Heat, Drought, Salt	↑	*Agrostisstolonifera L*	AsAFB2 and AsTIR1	Enhanced heat stress tolerance due to induced expression of small heat-shock protein under drought stress showed fewer, longer tillers, enhanced tolerance due to reduced stomatal density and denser cuticles.	[193]
					Conferred salt stress tolerance by increased uptake of potassium	
miR398	copper sulfate stress	↑	*Arabidopsis thaliana*	Copper/Zinc superoxide dismutases (CSDs) cytosolic (CSD1) andchloroplastic (CSD2) and 5b subunit of mitochondrial cytochrome C oxidase	reduction in root length and cotyledon greening, decreased expression of CSD1, CSD2, and CSD3. Increased production of superoxide dismutase. Decreases tolerance against copper sulfate stress and hence a negative regulator	[194]
				(COX5b.1)		
miR166	Cadmium stress	↑	*Oryza sativa*	class-III homeodomain-Leu zipper (HD-Zip) family proteins	improved Cd tolerance by decreasing Cd-induced oxidative stress. Reduced Cd translocation from roots to shoots	[163]
miR163	Light	↑	*Arabidopsis thaliana*	Long Hypocotyl 5 (HY5), PXMT1	Enhanced primary root elongation without hampering the lateral root growth in a light dependent manner. Contributes positively in regulation of root photomorphogenesis mediated by the HY5-miR163-PXMT1 network	[157]
miR828	Light	↓	*Brassica rapa*	BrPAP1, BrMYB82, and BrTAS4	light-induced down-regulation of BrmiR828 can negatively regulate the target transcript levels leading to the accumulation of MYB transcription factors that positively regulate anthocyanin biosynthesis in light-exposed seedlings of *Brassica rapa*.	[156]
OsmiR156k	cold	↑	*Oryza Sativa*	SPL3, SPL14 and SPL17	Decreased plant cold tolerance at the young seedling growth stage, as evidenced by lower survival rates, chlorophyll contents proline contents and inhibit the seedling growth at the very early seedling stage under cold stress.	[195]
Musa-miR397	Salt, Copper stress	↓&↑	*Musa spp.*	Laccases, WRKYs, E3 ubiquitin ligases PUB19,F-box/kelch repeat protein, DNAJ8, ABCG40 and cytochrome b561	Overexpression in banana plants significantly enhanced plant growth and tolerance toward Cu deficit and salt stress	[190]
miR398	Heat	↑&↓	*Arabidopsis thaliana*	CSD1, CSD2,and CCS	Knock down of NAT398b and NAT398c promotes miR398 processing, resulting in stronger plant thermotolerance owing to silencing of miR398-targeted genes; in contrast, their overexpression activates NAT398b and NAT398c, causing poorer thermotolerance due to the upregulation of miR398-targeted genes.	[196]
**Combined environmental stresses**						
miR156	Salt and drought	↑	*NicotianaTabaccum*		Positive regulator	[173]
miR172c	Water deficit and salt tolerance	↑	*Arabidopsis thaliana*		Positive regulator	[174]
miR535	Salt, drought, PEG and ABA	↓	*Oryza sativa*		Negative regulator	[153]

↑: Overexpression; ↓: knockdown.

## 7. Conclusion and future prospects

Climate change and environmental challenges severely hamper the crop production and thus global food security. As a common approach, the crops require an immediate yet effective genetic (or epigenetic) upgrade to thrive well and give better yields and vigor in the predicted challenging environmental conditions. Toward this, emphasis has been laid heavily on understanding and exploring the role of effective regulators of plant responses to stress factors, and the miRNAs are emerging as key signaling regulators for their differential gene expression in response to single/multiple/combined stress conditions. Thus, the target is to exploit the miRNAs/mRNAs and their potential interference in diverse cellular and molecular signaling cascades by utilizing genomics and other biotechnological tools. Recent developments in bioinformatics have enabled the identification, characterization and validation of data regarding miRNAs and their role in stress responses. Several microRNAs including miR408, miR156, miR393 are involved in various abiotic stresses. Additionally, considering the fact that plants suffer combined stresses in their native habitat, three miRNAs namely; miR156, miR172c and miR535 are shown to be involved conferring tolerance against multiple stress (Salt + drought, Water deficit + salt and Salt + drought + PEG + ABA) respectively. There is a renewed interest on finding the cross-talk associated microRNAs for combinational stresses, for example miRNAs responsive to CO2 and light stress. This opens up a new arena for research that needs to be explored to establish the data on miRNAs responsive to combination of stress factors. The algorithms established by bioinformatics researchers have paved the way for machine learning that has made studies on miRNAs faster, accurate and much more efficient. The advances in genome editing like CRISPR/cas9 have been highlighted for producing transgenic crops that are bioengineered to sustain harsh conditions. Techniques like artificial miRNA, topical delivery and target mimicry have been discussed as a means for crop improvement.

To mimic the changing climate conditions, researchers need to change their outlook in developing a single stress tolerant/resistant crop to the multiple stress tolerant crops. The signaling routes involved in modulating the miRNA directed multiple target genes should also be identified. A smart research shift is required wherein current artificial intelligence and machine learning based programs can be developed and explored for empowering the looming crop improvement. Broader applicability of the miRNA-based technology, public awareness about the dynamic climate change and linked practices, funding from the government bodies and landmark discoveries by the scientific community are the suggested steps to create miRNA based futuristic smart crops and a safe agro-ecosystem for all. The thrust of 3S- ‘SAFETY, SECURITY and SUSTAINABILITY’ activities happening globally are the obvious indications that mankind has accepted the interconnected impending climate changes and SDGs and is thus making themselves future ready by optimal utilization of tools leading to the development of miRNA-bioengineered climate resilient crops.
